# Deaths Mentioning Both Pancreatic Cancer and Renal Failure in the United States, 1999–2024: Temporal Trends and Disparities

**DOI:** 10.3390/epidemiologia7050124

**Published:** 2026-09-04

**Authors:** Arkadeep Dhali, Ali Shan Hafeez, Jyotirmoy Biswas, Ashish Sharma, Dushyant Singh Dahiya, Rick Maity, Saikat Mandal

**Affiliations:** 1 Academic Unit of Gastroenterology, Sheffield Teaching Hospitals NHS Foundation Trust, Sheffield S5 7AU, UK; 2 School of Medicine, Dentistry and Biomedical Sciences, Queen’s University of Belfast, Belfast BT9 7BL, UK; 3 Department of General Medicine, CMH Multan Institute of Medical Sciences, Multan 6000, Pakistan; 4 Department of General Medicine, Barasat Government Medical College and Hospital, Barasat 700124, India; 5 Department of Hospital Medicine, Yale New Haven Hospital, New Haven, CT 06510, USA; 6 Division of Gastroenterology, Hepatology & Motility, The University of Kansas School of Medicine, Kansas City, KS 66160, USA; 7 School of Medical Science and Technology, Indian Institute of Technology Kharagpur, Kharagpur 721302, India; 8 School of Medicine, University of Nottingham, Nottingham NG7 2UH, UK

**Keywords:** pancreatic cancer, renal failure, acute kidney failure, chronic kidney disease, multiple causes of death, mortality, health disparities, CDC WONDER, joinpoint regression

## Abstract

Background: We examined temporal trends in deaths mentioning both pancreatic cancer and renal failure in the United States. Methods: We analyzed CDC WONDER multiple-cause-of-death data for adults aged ≥25 years from 1999 to 2024. Age-adjusted mortality rates (AAMRs) were assessed using Joinpoint regression. Secondary analyses quantified renal-failure co-recording among pancreatic-cancer-involving deaths, renal-failure subtypes, disparities, and comparisons with lung and bronchus, colorectal, and liver and intrahepatic bile duct cancers. Results: Overall, 31,497 deaths mentioned both conditions. The AAMR increased from 0.37 per 100,000 in 1999 to 0.82 in 2024, with the most rapid increase during 2018–2024 (annual percentage change, 10.77%; 95% CI, 7.18–18.22%). Renal failure was co-recorded in 2.98% of pancreatic-cancer-involving deaths. Corresponding proportions were 2.37% for lung and bronchus, 4.75% for colorectal, and 5.77% for liver and intrahepatic bile duct cancer; recent trends did not differ significantly from pancreatic cancer. Acute kidney failure was the most frequently recorded renal subtype (43.22%). In 2024, AAMRs were higher among males and highest among non-Hispanic Black individuals. Conclusions: Renal-failure co-recording with pancreatic cancer increased markedly after 2018 but was not unique to pancreatic cancer. Death-certificate data do not establish temporal sequence or causality; linked clinical data are needed to clarify potentially modifiable kidney-related pathways.

## 1. Introduction

Pancreatic cancer remains one of the most lethal gastrointestinal malignancies, with mortality increasing in the United States. A national analysis of deaths from 1999 to 2023 demonstrated rising age-adjusted pancreatic-cancer mortality together with differences by sex, race and ethnicity, region, and urbanization [1]. These findings establish the substantial population burden of pancreatic cancer but provide limited information about other clinically important conditions recorded at the time of death.

Kidney dysfunction is common and prognostically relevant in oncology populations. Renal failure is a heterogeneous clinical category encompassing acute kidney failure, chronic kidney disease, and unspecified kidney failure. In a large oncology cohort, chronic kidney disease stages G3–G5 were identified in 19.6% of patients with pancreatic cancer [2]. A nationwide Korean cohort found pancreatic cancer to occur more frequently among patients with pre-dialytic chronic kidney disease than in the comparison population [3]. Among 2323 patients with pancreatic cancer, reduced kidney function at diagnosis was associated with poorer overall survival [4]. Chronic kidney disease severity has also been associated with postoperative complications and mortality after pancreatic resection for malignancy [5]. These studies demonstrate the clinical relevance of kidney dysfunction among patients with pancreatic cancer but do not describe population-level deaths in which both conditions were recorded on the same death certificate.

Multiple-cause-of-death data capture the underlying cause of death together with other conditions recorded as contributing causes. A mortality rate based on death certificates mentioning both pancreatic cancer and renal failure therefore measures the population frequency of deaths in which both conditions were co-recorded. This measure complements, rather than replaces, condition-specific mortality rates by identifying the subset of deaths in which both diagnoses were documented by the death certifier. It does not estimate renal-failure incidence among patients with pancreatic cancer, mortality risk within a pancreatic-cancer cohort, the temporal order of the two conditions, or the causal contribution of renal failure to death. Its epidemiologic value is to quantify and characterize the population distribution of documented co-occurrence. Whether renal failure is co-recorded more frequently or has increased more rapidly in pancreatic-cancer-involving deaths than in deaths involving other major cancers is also uncertain.

The frequency, temporal pattern, and demographic distribution of deaths mentioning both pancreatic cancer and renal failure have not been comprehensively described in the United States. We therefore examined mortality involving co-recording of both conditions from 1999 through 2024. Our objectives were to: (1) characterize nonlinear temporal trends in population-level mortality involving both conditions; (2) quantify the percentage of pancreatic-cancer-involving deaths that also mentioned renal failure; (3) examine acute kidney failure, chronic kidney disease, and unspecified kidney failure separately; (4) investigate the transient elevation around 2011–2012; (5) evaluate differences by sex, race and ethnicity, Census region, state, and urbanization; and (6) compare renal-failure co-recording with other selected major cancers to assess whether the observed recent increase was specific to pancreatic-cancer-involving deaths or reflected a broader pattern across cancer mortality.

## 2. Methods

### 2.1. Study Design and Data Source

This was a retrospective, population-based mortality study using final data from the Centers for Disease Control and Prevention Wide-ranging Online Data for Epidemiologic Research Multiple Cause of Death database [6]. Data were extracted on 5–6 June 2026. Estimates for 1999–2020 were obtained from the Multiple Cause of Death, 1999–2020 dataset, and estimates for 2021–2024 were obtained from the Multiple Cause of Death, 2018–2024 Single Race dataset. Across the overlapping years 2018–2020, the two datasets produced identical death counts and AAMRs for the overall, sex, Census-region, pancreatic-cancer denominator, and renal-subtype series. Query parameters selected decedents aged ≥25 years, used the 2000 United States standard population for direct age adjustment, and calculated rates per 100,000 population. Because the data were deidentified, publicly available, and aggregate-level, institutional review board approval and informed consent were not required. The study followed STROBE reporting guidance [7].

For the primary co-recorded outcome, the CDC WONDER query required at least one pancreatic-cancer code and at least one renal-failure code to be recorded anywhere among the multiple-cause fields. A separate pancreatic-cancer-only query was used to obtain the denominator for the percentage of pancreatic-cancer-involving deaths that also mentioned renal failure. Additional queries combined the pancreatic-cancer codes separately with acute kidney failure, chronic kidney disease, and unspecified kidney failure. Query outputs were grouped by year and, where applicable, by sex, race and ethnicity, Census region, state, and urbanization. The same dataset-combination rule was applied to all queries: estimates for 1999–2020 were retained from the earlier dataset and estimates for 2021–2024 from the expanded Single Race dataset.

For a post hoc cancer-comparator analysis, analogous paired queries were performed for lung and bronchus cancer (ICD-10 C34), colorectal cancer (C18–C20), and liver and intrahepatic bile duct cancer (C22), both with and without renal failure (N17–N19). These cancers were selected as major solid-tumor comparators, including gastrointestinal and non-gastrointestinal malignancies. The same age restriction, multiple-cause definition, annual grouping, and dataset-combination rule used for the pancreatic-cancer analysis were applied to all comparator queries.

### 2.2. Case Definition

A co-recorded death was defined as a death certificate on which both pancreatic cancer and renal failure were listed anywhere among the underlying or contributing causes of death. Each included death represented one decedent, regardless of the number of qualifying pancreatic-cancer or renal-failure codes recorded.

Pancreatic cancer was identified using ICD-10 codes C25.0, C25.1, C25.2, C25.3, C25.4, C25.7, C25.8, and C25.9. Renal failure was identified using ICD-10 codes N17–N19. Acute kidney failure included N17.0, N17.1, N17.2, N17.8, and N17.9. Chronic kidney disease included N18.0, N18.1, N18.2, N18.3, N18.4, N18.5, N18.8, and N18.9. Unspecified kidney failure was identified using N19. Because more than one renal-failure code could appear on the same death certificate, subtype-specific any-mention categories were not mutually exclusive.

The analytic period extended from January 1999 through December 2024. Urbanization-stratified analyses were limited to 1999–2020 because harmonized urbanization extracts were included only for this period in the analytic dataset.

### 2.3. Study Variables and Outcomes

The study population included United States decedents aged 25 years or older. Mortality was analyzed by year of death, sex, race and ethnicity, United States Census region, state, and urbanization status.

Sex was categorized as female or male. Race and ethnicity were categorized as non-Hispanic White, non-Hispanic Black or African American, and Hispanic or Latino. The 1999–2020 non-Hispanic race estimates were obtained using the bridged-race categories in the earlier CDC WONDER dataset, whereas the 2021–2024 estimates were obtained using the Single Race 6 categories in the expanded dataset. Census regions were defined as Northeast, Midwest, South, and West. Urbanization was classified using the National Center for Health Statistics Urban–Rural Classification Scheme for Counties and grouped as metropolitan or nonmetropolitan.

The primary outcome was the annual age-adjusted mortality rate per 100,000 population for deaths mentioning both pancreatic cancer and renal failure. Rates were standardized to the 2000 United States standard population using the direct method [8].

The principal secondary outcome was the annual percentage of pancreatic-cancer-involving deaths that also mentioned renal failure. This percentage was calculated by dividing the number of deaths mentioning both pancreatic cancer and renal failure by the number of deaths mentioning pancreatic cancer anywhere on the death certificate and multiplying by 100.

Additional secondary outcomes included crude mortality rates, absolute death counts, renal-failure subtype distributions, subgroup-specific mortality patterns, state-level variation, and year-specific changes surrounding 2011–2012.

### 2.4. Statistical Analysis

Temporal trends in age-adjusted mortality rates were analyzed using the Joinpoint Regression Program, version 6.0.1. Joinpoint regression estimated annual percent changes and average annual percent changes with 95% confidence intervals. Statistical significance was assessed using permutation-based model selection, with a two-sided *p* value < 0.05 considered statistically significant.

Annual co-recording percentages were calculated by dividing the number of deaths mentioning both pancreatic cancer and renal failure by the number of deaths mentioning pancreatic cancer anywhere on the death certificate. Percentages were presented with 95% confidence intervals calculated using the Wilson score method without continuity correction. Renal-subtype analyses were descriptive and included total counts, percentages of all co-recorded deaths, annual counts, and annual AAMRs. Because subtype-specific any-mention categories could overlap, their percentages were not expected to sum to 100%.

For each comparator cancer, the annual renal-failure co-recording percentage was calculated as the number of deaths mentioning both that cancer and renal failure divided by the number of deaths mentioning that cancer anywhere on the death certificate. Overall percentages and Wilson 95% confidence intervals were calculated using the same approach as for pancreatic cancer. Because 2018 marked the beginning of the most recent increasing segment in the primary pancreatic-cancer analysis, recent trends were compared over a common fixed period of 2018–2024. For each cancer group, log-linear regression of the annual co-recording percentage on calendar year was used to estimate the annual percent change and 95% confidence interval during this fixed period. A pooled log-linear model including cancer group, year, and cancer-by-year interaction terms was used to test whether each comparator trend differed from the pancreatic-cancer trend. These comparator analyses were considered secondary and descriptive.

Data management and descriptive analyses were conducted using R version 4.6.0 (R Foundation for Statistical Computing, Vienna, Austria). Joinpoint analyses were performed using the Joinpoint Regression Program, version 6.0.1.

The transient elevation around 2011–2012 was investigated by rechecking the original CDC WONDER exports and cleaned analytic datasets; comparing annual death counts, crude rates, AAMRs, and co-recording percentages for 2009–2014; and examining sex-, Census-region-, and renal-subtype-specific series. Year-to-year percentage changes were calculated, and a three-year centered moving average was used as a descriptive sensitivity analysis. The observed unsmoothed annual series remained the primary analysis.

## 3. Results

### 3.1. Overall Mortality Trends

From 1999 to 2024, 31,497 deaths among United States adults aged ≥25 years mentioned both pancreatic cancer and renal failure. Annual co-recorded deaths increased from 689 in 1999 to 2365 in 2024, while the age-adjusted mortality rate increased from 0.37 per 100,000 to 0.82 per 100,000.

**Figure 1:**
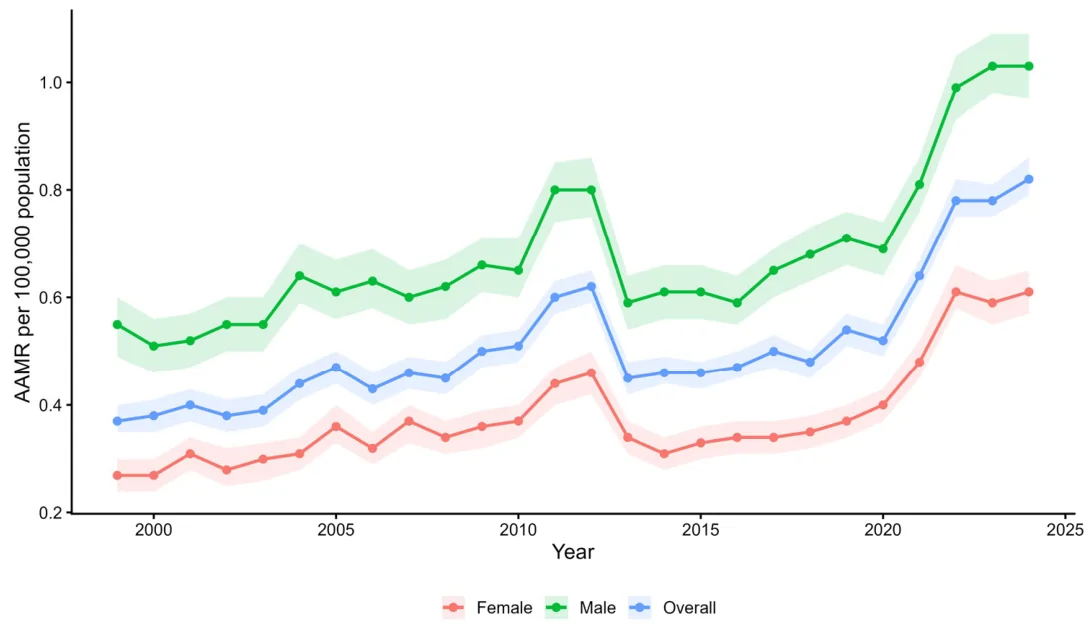
Age-adjusted mortality rates per 100,000 population for deaths mentioning both pancreatic cancer and renal failure, overall and by sex, United States, 1999–2024. Shaded areas represent 95% confidence intervals. Detailed Joinpoint estimates are presented in Supplementary Table S1. AAMR, age-adjusted mortality rate.

The temporal pattern was nonlinear rather than continuously increasing. Joinpoint regression identified two inflection points. Age-adjusted mortality rates (Figure 1, Table 1) increased significantly from 1999 to 2011 (APC, 2.90%; 95% CI, 1.70–9.17%), did not change significantly from 2011 to 2018 (APC, −1.46%; 95% CI, −8.34% to 1.06%), and increased sharply from 2018 to 2024 (APC, 10.77%; 95% CI, 7.18–18.22%). Across the full study period, mortality increased significantly (AAPC, 3.47%; 95% CI, 2.89–4.23%).

### 3.2. Frequency of Renal-Failure Recording and Renal-Failure Subtypes

During the full study period, renal failure was recorded in 31,497 of 1,057,018 pancreatic-cancer-involving deaths (2.98%; 95% CI, 2.95–3.01%). The annual percentage increased from 2.26% (95% CI, 2.10–2.43%) in 1999 to 4.33% (95% CI, 4.17–4.51%) in 2024. The pattern was nonlinear: the percentage increased transiently to 3.41% in 2011 and 3.44% in 2012, declined to 2.61% in 2013, and subsequently increased, particularly after 2020.

Among the 31,497 deaths mentioning both pancreatic cancer and renal failure, 13,612 (43.22%; 95% CI, 42.67–43.76%) mentioned acute kidney failure, 8818 (28.00%; 95% CI, 27.50–28.49%) mentioned chronic kidney disease, and 9728 (30.89%; 95% CI, 30.38–31.40%) mentioned unspecified kidney failure. These any-mention categories were not mutually exclusive because an individual death certificate could include more than one renal-failure subtype. Acute kidney failure was the most frequently recorded subtype and increased from an AAMR of 0.09 per 100,000 in 1999 to 0.52 in 2024. The chronic kidney disease AAMR increased from 0.07 to 0.23, whereas the unspecified kidney failure AAMR declined from 0.23 to 0.08. Detailed results are presented in Table 2 and Supplementary Figure S1.

### 3.3. Comparison with Other Major Cancers

During 1999–2024, renal failure was co-recorded in 31,497 of 1,057,018 pancreatic-cancer-involving deaths (2.98%; 95% CI, 2.95–3.01%). The corresponding frequencies were 99,100 of 4,182,597 lung and bronchus cancer-involving deaths (2.37%; 95% CI, 2.35–2.38%), 76,684 of 1,615,156 colorectal cancer-involving deaths (4.75%; 95% CI, 4.72–4.78%), and 35,676 of 618,504 liver and intrahepatic bile duct cancer-involving deaths (5.77%; 95% CI, 5.71–5.83%). Thus, the overall frequency of renal-failure co-recording in pancreatic-cancer-involving deaths was higher than that observed for lung and bronchus cancer but lower than that observed for colorectal and liver and intrahepatic bile duct cancers.

From 2018 to 2024, the renal-failure co-recording percentage increased from 2.77% to 4.33% for pancreatic cancer, from 2.38% to 3.70% for lung and bronchus cancer, from 4.34% to 6.04% for colorectal cancer, and from 5.42% to 7.88% for liver and intrahepatic bile duct cancer. Fixed-period annual percent changes during 2018–2024 were 9.15% (95% CI, 5.72–12.68%) for pancreatic cancer, 8.91% (95% CI, 5.68–12.23%) for lung and bronchus cancer, 6.96% (95% CI, 4.39–9.59%) for colorectal cancer, and 7.79% (95% CI, 4.84–10.83%) for liver and intrahepatic bile duct cancer. Compared with the pancreatic-cancer trend, differences in the recent slopes were not statistically significant for lung and bronchus cancer (*p* = 0.892), colorectal cancer (*p* = 0.214), or liver and intrahepatic bile duct cancer (*p* = 0.438). These findings indicate that the recent increase in renal-failure co-recording was not unique to pancreatic cancer. Detailed comparator results are presented in Supplementary Table S3.

### 3.4. Investigation of the 2011–2012 Temporal Pattern

The transient elevation around 2011–2012 was confirmed in the original CDC WONDER exports and cleaned analytic dataset. Annual co-recorded deaths increased from 1096 in 2010 to 1327 in 2011, representing a 21.1% year-to-year increase, and to 1388 in 2012, representing a further 4.6% increase. Deaths subsequently declined to 1062 in 2013, representing a 23.5% reduction from 2012. Corresponding AAMRs were 0.51, 0.60, 0.62, and 0.45 per 100,000, respectively. The percentages of pancreatic-cancer-involving deaths that also mentioned renal failure were 2.85% in 2010, 3.41% in 2011, 3.44% in 2012, and 2.61% in 2013, confirming that the elevation was not explained solely by growth in pancreatic-cancer-involving deaths.

The elevation followed by decline was observed among both sexes and across all four Census regions (Supplementary Table S2). Renal-subtype analyses showed that chronic kidney disease mentions increased from 225 deaths in 2010 (AAMR, 0.10) to 431 in 2011 (AAMR, 0.18) and 492 in 2012 (AAMR, 0.22), before declining to 300 in 2013 (AAMR, 0.12). Acute kidney failure AAMRs remained comparatively similar at 0.19, 0.20, 0.19, and 0.21 in 2010–2013, respectively. Unspecified kidney failure AAMRs were 0.22, 0.21, 0.22, and 0.13, respectively. Thus, the transient 2011–2012 elevation was most apparent in chronic kidney disease recording, while the 2013 decline coincided with reductions in chronic kidney disease and unspecified kidney failure recording. Because the subtype categories could overlap, these changes should be interpreted descriptively. The three-year centered moving-average AAMRs were 0.54, 0.58, 0.56, and 0.51 in 2010–2013, respectively. Re-examination identified no arithmetic, denominator, duplication, or dataset-stitching error. The aggregate data could not determine whether the pattern reflected a temporary epidemiologic change or changes in diagnostic and death-certificate recording practices.

### 3.5. Sex-Specific Trends

AAMRs were higher among males than females throughout the study period. From 1999 to 2024, AAMRs increased from 0.55 to 1.03 per 100,000 among males and from 0.27 to 0.61 among females. Overall, 18,281 deaths occurred among males and 13,216 among females. Among females, AAMRs increased during 1999–2012 (APC, 3.74%; 95% CI, 2.58–6.33%), declined during 2012–2015 (APC, −12.21%; 95% CI, −16.63% to −2.62%), and increased during 2015–2024 (APC, 9.21%; 95% CI, 7.42–13.13%). Among males, AAMRs increased during 1999–2012 (APC, 2.84%; 95% CI, 1.79–5.38%), declined during 2012–2016 (APC, −6.28%; 95% CI, −11.68% to −1.30%), and increased during 2016–2024 (APC, 8.15%; 95% CI, 6.38–11.48%). Full-period AAPCs were 3.58% (95% CI, 3.13–4.45%) among females and 2.97% (95% CI, 2.55–3.70%) among males.

### 3.6. Race- and Ethnicity-Specific Trends

**Figure 2:**
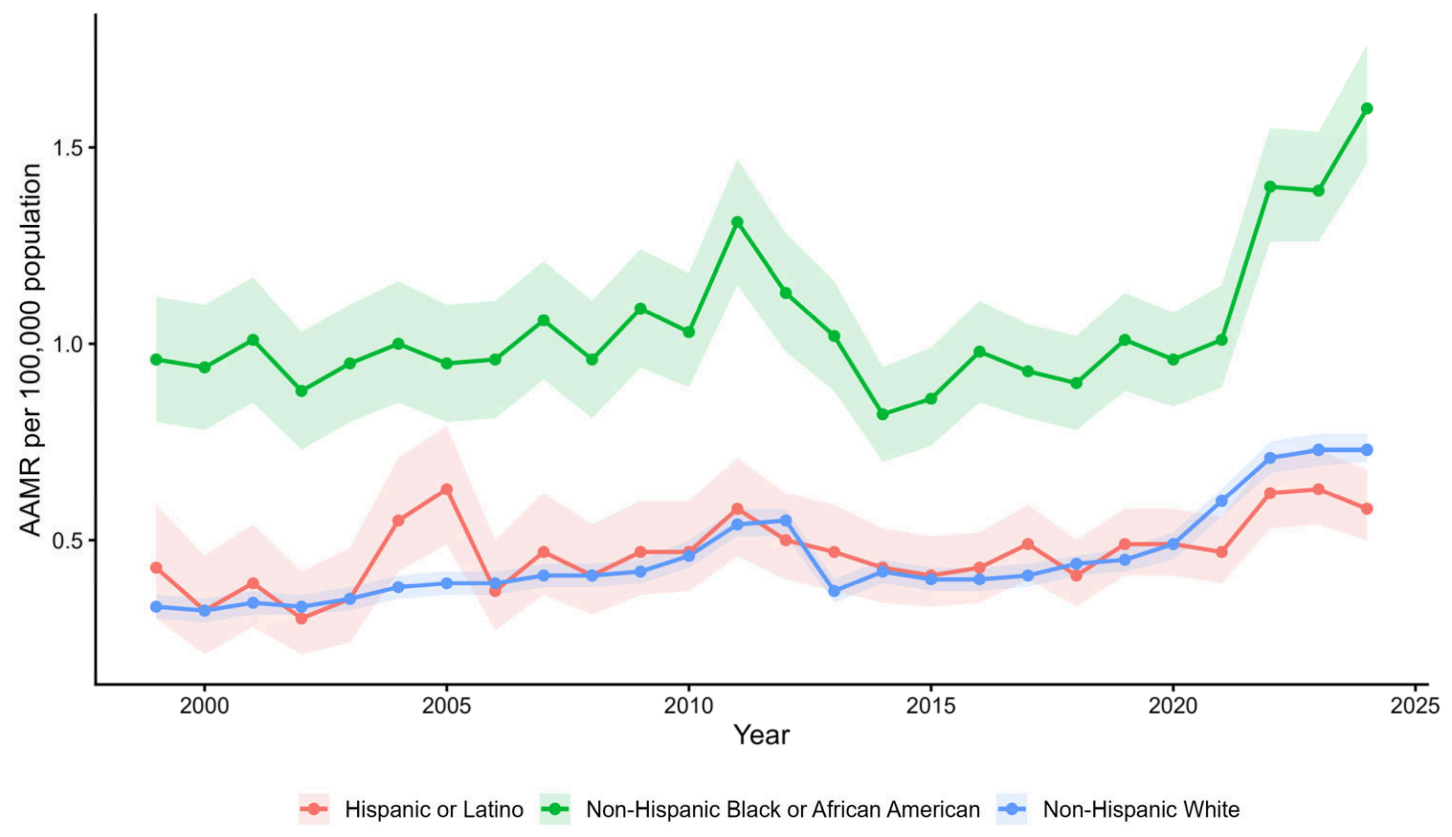
Age-adjusted mortality rates per 100,000 population for deaths mentioning both pancreatic cancer and renal failure, by race and ethnicity, United States, 1999–2024. Shaded areas represent 95% confidence intervals. Detailed Joinpoint estimates are presented in Supplementary Table S1. AAMR, age-adjusted mortality rate.

Substantial differences in AAMRs were observed by race and ethnicity (Figure 2). Non-Hispanic Black or African American individuals had the highest AAMRs throughout the study period, increasing from 0.96 per 100,000 in 1999 to 1.60 in 2024. AAMRs increased from 0.33 to 0.73 among non-Hispanic White individuals and from 0.43 to 0.58 among Hispanic or Latino individuals. Among non-Hispanic Black or African American individuals, the most rapid increase occurred during 2020–2024 (APC, 13.32%; 95% CI, 8.78–22.04%). Among non-Hispanic White individuals, AAMRs increased during 1999–2011 (APC, 3.91%; 95% CI, 2.53–6.89%), declined during 2011–2016 (APC, −5.18%; 95% CI, −12.46% to −0.93%), and increased during 2016–2024 (APC, 9.50%; 95% CI, 7.50–12.41%). Hispanic or Latino individuals had a linear increase during 1999–2024 (APC, 1.34%; 95% CI, 0.45–2.55%). Full-period AAPCs were 2.15% (95% CI, 1.57–2.78%) among non-Hispanic Black or African American individuals, 3.75% (95% CI, 3.26–4.42%) among non-Hispanic White individuals, and 1.34% (95% CI, 0.45–2.55%) among Hispanic or Latino individuals.

### 3.7. Regional Trends

**Figure 3:**
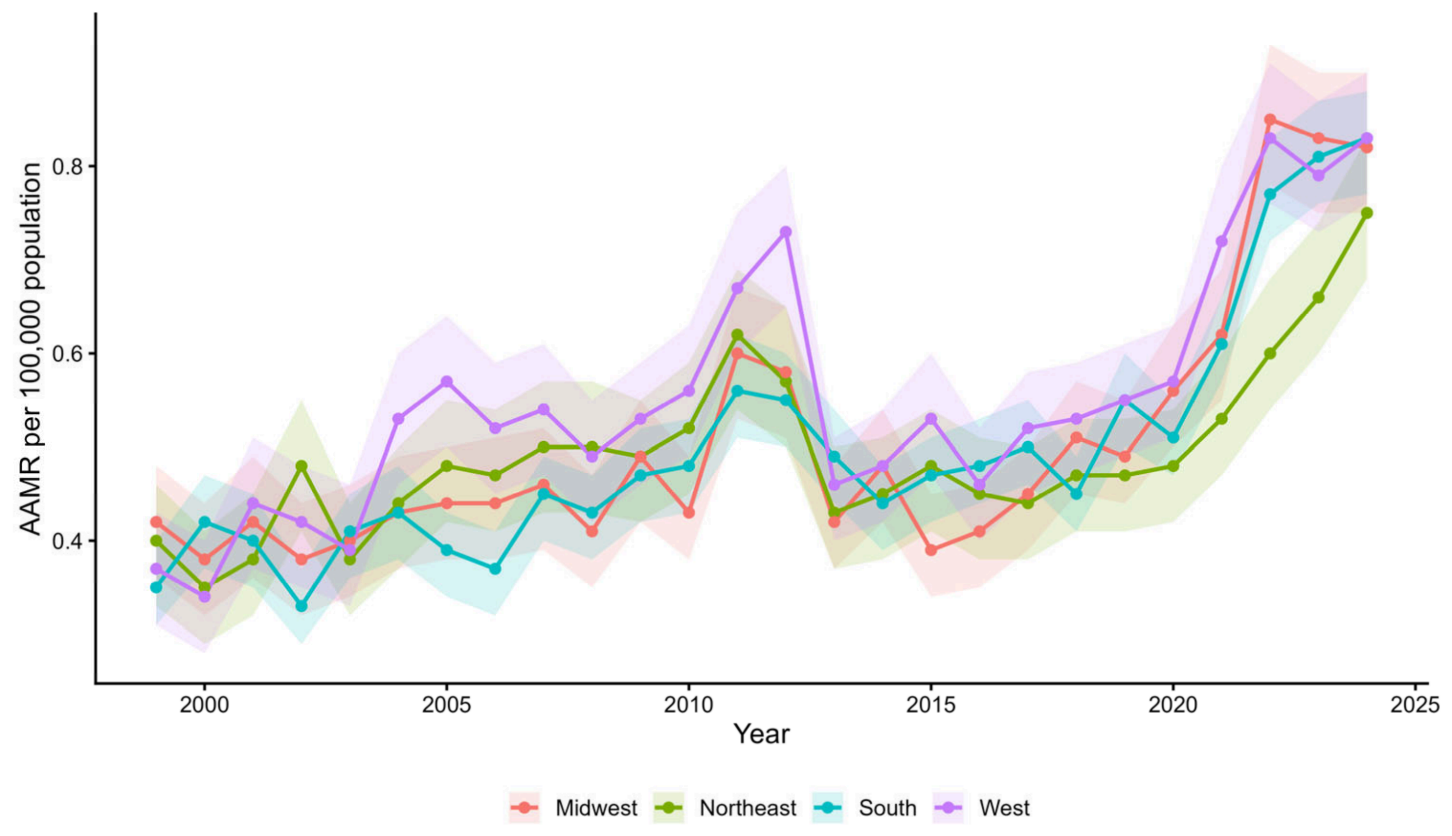
Age-adjusted mortality rates per 100,000 population for deaths mentioning both pancreatic cancer and renal failure, by United States Census region, 1999–2024. Shaded areas represent 95% confidence intervals. Detailed Joinpoint estimates are presented in Supplementary Table S1. AAMR, age-adjusted mortality rate.

AAMRs increased significantly across all United States Census regions (Figure 3). In 2024, the highest AAMRs were observed in the South and West, both at 0.83 per 100,000, followed by the Midwest at 0.82 and the Northeast at 0.75. Across the full study period, the South accounted for the largest number of deaths (11,457), followed by the West (7411), Midwest (6871), and Northeast (5758). Full-period AAPCs were 3.53% (95% CI, 2.75–4.22%) in the South, 3.49% (95% CI, 2.90–4.53%) in the Midwest, 3.43% (95% CI, 2.77–4.67%) in the West, and 2.60% (95% CI, 2.03–3.50%) in the Northeast. Detailed segment-specific results are presented in Supplementary Table S1.

### 3.8. Urbanization Trends

**Figure 4:**
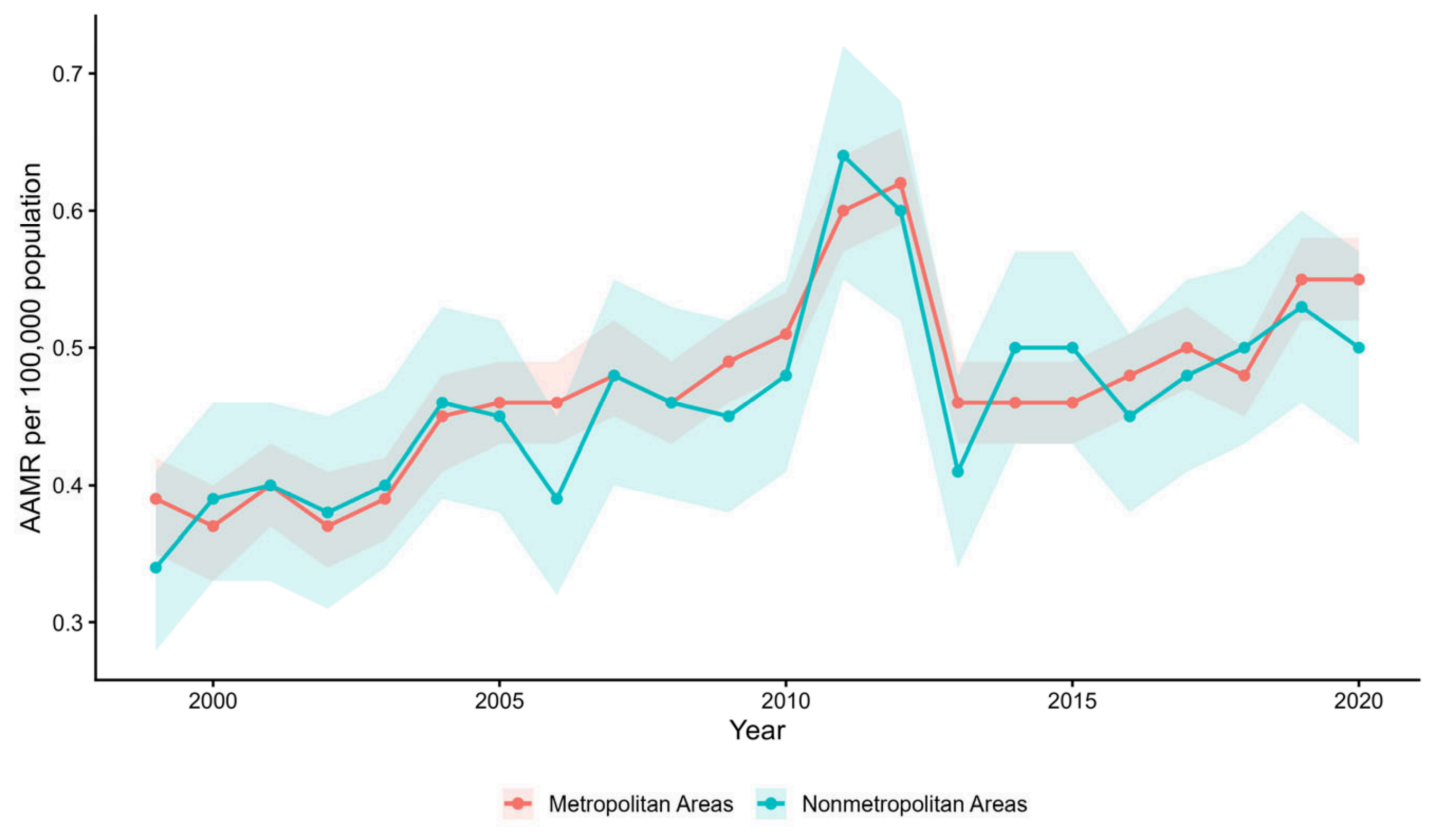
Age-adjusted mortality rates per 100,000 population for deaths mentioning both pancreatic cancer and renal failure, by urbanization, United States, 1999–2020. Shaded areas represent 95% confidence intervals. Detailed Joinpoint estimates are presented in Supplementary Table S1. AAMR, age-adjusted mortality rate.

Urbanization analyses were limited to 1999–2020. AAMRs increased from 0.39 to 0.55 per 100,000 in metropolitan areas and from 0.34 to 0.50 in nonmetropolitan areas (Figure 4). During this period, 19,097 deaths occurred in metropolitan areas and 3927 in nonmetropolitan areas. The full-period AAPC was 2.00% (95% CI, 1.09–2.95%) in metropolitan areas and 1.46% (95% CI, 0.40–2.87%) in nonmetropolitan areas. Among nonmetropolitan areas, AAMRs increased during 1999–2011 (APC, 3.28%; 95% CI, 1.76–15.54%) and did not change significantly during 2011–2020 (APC, −0.93%; 95% CI, −9.13% to 1.24%). Metropolitan segment-specific changes were not statistically significant, although the full-period AAPC was significant.

### 3.9. State-Level Variation

**Figure 5:**
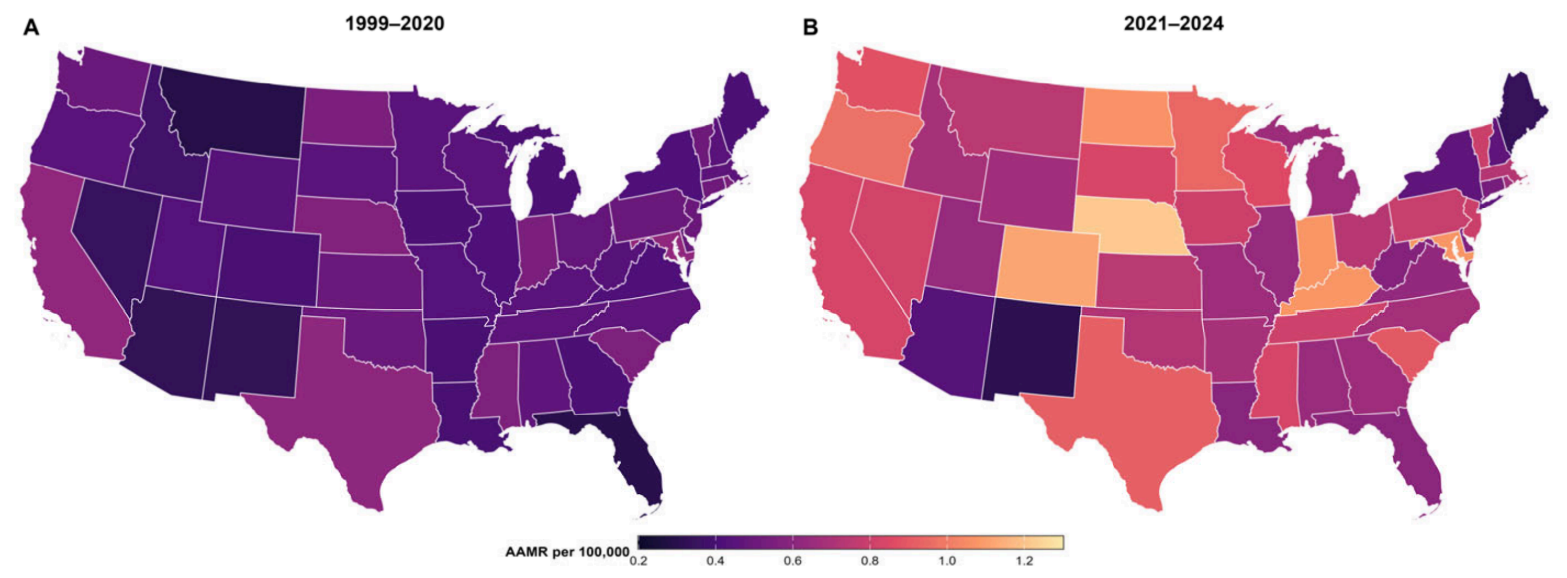
State- and District of Columbia-level age-adjusted mortality rates per 100,000 population for deaths mentioning both pancreatic cancer and renal failure during (**A**) 1999–2020 and (**B**) 2021–2024. The panels summarize periods of different durations and should not be interpreted as direct before-and-after comparisons. AAMR, age-adjusted mortality rate.

State-level AAMRs varied substantially (Figure 5). During 1999–2020, the highest AAMRs were observed in the District of Columbia at 0.80 per 100,000, followed by California and Maryland, both at 0.62, and Texas at 0.61. The lowest AAMRs were observed in Montana at 0.29 per 100,000, Florida at 0.30, and Arizona and New Mexico, both at 0.33. During 2021–2024, the highest AAMRs were observed in Nebraska at 1.22 per 100,000, the District of Columbia at 1.15, Colorado at 1.12, and Indiana, Kentucky, and Maryland, each at 1.08. The lowest recent AAMRs were observed in New Mexico at 0.31 per 100,000, Maine at 0.34, and Arizona at 0.44.

## 4. Discussion

In this nationwide multiple-cause-of-death analysis, 31,497 of 1,057,018 pancreatic-cancer-involving deaths among United States adults aged ≥25 years also mentioned renal failure between 1999 and 2024. Thus, renal failure was co-recorded in 2.98% of pancreatic-cancer-involving deaths. The AAMR increased from 0.37 to 0.82 per 100,000, but the temporal pattern was nonlinear, comprising an initial increase, no statistically significant change during 2011–2018, and a marked increase after 2018. AAMRs were higher among males, highest among non-Hispanic Black individuals, and varied across regions and states.

The outcome examined in this study represents the population frequency of death certificates on which both pancreatic cancer and renal failure were recorded. It complements condition-specific mortality statistics by identifying deaths in which both diagnoses were considered sufficiently relevant to be documented by the death certifier. The pancreatic-cancer-specific denominator provides additional context: although the population AAMR for co-recorded deaths increased, these deaths accounted for 2.98% of all pancreatic-cancer-involving deaths during the full study period. Nevertheless, the outcome does not estimate renal-failure incidence among patients with pancreatic cancer, mortality risk within a pancreatic-cancer cohort, the temporal sequence of the two conditions, or the causal contribution of renal failure to death.

The comparator analysis provides important context for the pancreatic-cancer findings. Renal failure was co-recorded in 2.98% of pancreatic-cancer-involving deaths, a frequency higher than that observed for lung and bronchus cancer but lower than those observed for colorectal and liver and intrahepatic bile duct cancers. Moreover, renal-failure co-recording increased during 2018–2024 across all four cancer groups, and the recent pancreatic-cancer trend did not differ significantly from any of the comparator trends. The increase observed in pancreatic-cancer-involving deaths should therefore not be interpreted as a uniquely pancreatic-cancer-specific phenomenon. Rather, it may partly reflect broader changes affecting patients with cancer, including the burden of kidney disease, increasing complexity of cancer care, recognition of renal dysfunction, and death-certificate documentation. Nevertheless, characterizing the pancreatic-cancer population remains clinically relevant because the underlying renal subtypes, clinical circumstances, and potentially modifiable pathways may differ across cancer populations.

The renal-subtype findings provide additional clinical context for the overall temporal pattern. Acute kidney failure was the most frequently recorded subtype, appearing on 43.22% of co-recorded death certificates, and its AAMR increased from 0.09 per 100,000 in 1999 to 0.52 in 2024. The chronic kidney disease AAMR also increased from 0.07 to 0.23, whereas the unspecified kidney failure AAMR declined from 0.23 to 0.08. The increasing recording of acute kidney failure and chronic kidney disease alongside declining unspecified kidney failure is compatible with an increasing burden or recognition of clinically specified kidney dysfunction rather than an increase driven solely by nonspecific renal-failure coding. In patients with cancer, acute kidney injury may occur through patient-related, cancer-related, and treatment-related pathways, including volume depletion, infection or sepsis, treatment-associated nephrotoxicity, urinary obstruction, and acute illness superimposed on pre-existing chronic kidney disease [9,10]. Pancreatic surgery represents an additional relevant setting; postoperative acute kidney injury has been documented after pancreaticoduodenectomy for pancreatic ductal adenocarcinoma, with reduced preoperative kidney function among the identified risk factors [11]. However, the present death-certificate data contain no laboratory measurements, treatment exposures, timings of kidney dysfunction, or details of terminal illness and therefore cannot determine which of these mechanisms explains the observed increase or whether renal failure causally contributed to death.

The transient elevation around 2011–2012 should be distinguished from the later post-2018 increase. The percentage of pancreatic-cancer-involving deaths that also mentioned renal failure increased from 2.85% in 2010 to 3.41% in 2011 and 3.44% in 2012 before declining to 2.61% in 2013, indicating that the pattern was not explained solely by growth in pancreatic-cancer-involving deaths. Similar elevations followed by declines were observed among both sexes and all four Census regions. Subtype analysis showed that the elevation was most apparent for chronic kidney disease recording, while the subsequent decline coincided with decreases in chronic kidney disease and unspecified kidney failure recording. Rechecking the source exports and analytic files did not identify an arithmetic, denominator, duplication, or dataset-stitching error. Nevertheless, the aggregate data cannot determine whether the pattern represented a temporary epidemiologic change or a change in diagnostic recognition or death-certificate reporting.

The sharp increase after 2018 should therefore be interpreted as potentially reflecting a mixture of clinical and documentation processes rather than a single mechanism. Population aging may contribute to increasing absolute death counts but cannot by itself explain an increase in age-adjusted rates. Changes in the prevalence or recognition of kidney dysfunction, treatment intensity and complexity, acute illness among patients with advanced cancer, and death-certificate documentation may all contribute. The increasing phase began before 2020; therefore, pandemic-era factors cannot explain its onset, although they may have influenced later years. The parallel increases observed in the comparator cancers further argue against attributing the post-2018 pattern to a pancreatic-cancer-specific mechanism on the basis of these data alone.

The higher AAMRs observed among males and non-Hispanic Black individuals parallel previously reported differences in pancreatic-cancer mortality [1,12]. Prior studies have documented differences in surgical evaluation, resection, cancer-directed treatment, and survival [13,14,15]. However, the demographic differences observed in the present study may reflect variation in pancreatic-cancer burden, renal-failure burden, coexistence of the two conditions, treatment access, population characteristics, or death-certificate documentation. Because information on cancer stage, treatment, insurance, socioeconomic circumstances, and healthcare access was unavailable, this study cannot determine which mechanisms produced the observed differences.

Geographic variation may similarly reflect multiple mechanisms. Regional and state differences could arise from variation in population characteristics, pancreatic-cancer burden, kidney-disease prevalence, specialist access, treatment patterns, or death-certificate reporting. Previous evidence concerning rural and urban pancreatic-cancer outcomes has been mixed [16,17,18]. The present analysis cannot determine whether the geographic variation reflects clinical care, population composition, coexistence of the two conditions, or documentation practices. State estimates should also be interpreted cautiously because some may be affected by smaller numbers and statistical instability.

The clinical and public-health value of these findings lies in identifying kidney-related pathways that warrant more focused investigation rather than establishing a specific preventive intervention. Future clinical and linked-data studies should determine whether renal-failure co-recording is concentrated among patients with reduced baseline kidney function or pre-existing chronic kidney disease; whether acute kidney injury develops during pancreatic surgery, systemic anticancer therapy, dehydration, infection or sepsis, urinary obstruction, or exposure to potentially nephrotoxic agents; and whether renal dysfunction interrupts or limits cancer-directed treatment [9,10,11]. These findings therefore highlight the importance of studying baseline kidney-function assessment, renal-function monitoring during high-risk treatment and acute illness, attention to volume status and potentially nephrotoxic exposures, prompt recognition of reversible precipitants of acute kidney injury, and timely nephrology involvement when kidney dysfunction is severe, is persistent, or threatens cancer treatment. These should be regarded as clinically plausible and potentially modifiable pathways for further evaluation rather than preventive strategies proven by the present mortality analysis. Linked cancer-registry, hospitalization, claims, laboratory, treatment, and mortality data are needed to distinguish potentially preventable acute kidney injury from renal failure occurring as part of advanced or terminal illness.

This study has several strengths. It used nationwide mortality data covering 26 years and included both underlying and contributing causes of death. The analysis used age-standardized rates, Joinpoint regression to characterize nonlinear temporal patterns, a pancreatic-cancer-specific denominator, separate analyses of acute kidney failure, chronic kidney disease, and unspecified kidney failure, a targeted comparison with selected major solid cancers, extensive demographic and geographic stratification, and targeted sensitivity analyses of the 2011–2012 elevation.

This study also has important limitations. First, death certificates are susceptible to diagnostic omission, miscoding, and variation in certifier practices across time and jurisdictions. Second, co-recording cannot establish the temporal sequence of pancreatic cancer and renal failure or determine whether renal failure causally contributed to death. Third, although ICD-10 codes distinguish acute kidney failure, chronic kidney disease, and unspecified kidney failure, they do not provide clinical severity, laboratory-defined kidney function, dialysis status, reversibility, or treatment attribution. Fourth, subtype categories could overlap, preventing the percentages from being interpreted as a mutually exclusive etiologic distribution. Fifth, information on pancreatic-cancer histology, stage, treatment, surgery, comorbidities, socioeconomic circumstances, insurance, and healthcare access was unavailable. Sixth, the transition from bridged-race to single-race datasets may affect comparability, particularly for race- and ethnicity-specific analyses. Seventh, smaller subgroup and state estimates may be statistically unstable or suppressed, and urbanization analyses were available only through 2020. Eighth, the post hoc comparator analysis was limited to three selected major solid cancers and was intended to provide epidemiologic context rather than a comprehensive comparison across all malignancies. Finally, the findings are descriptive and should not be interpreted as estimates of individual-level risk.

## 5. Conclusions

Between 1999 and 2024, the United States AAMR for deaths mentioning both pancreatic cancer and renal failure followed a nonlinear pattern, with an initial increase, no statistically significant change during 2011–2018, and a marked increase after 2018. Renal failure was co-recorded in 2.98% of pancreatic-cancer-involving deaths, and acute kidney failure was the most frequently recorded renal subtype. Comparison with selected major cancers demonstrated that renal-failure co-recording was not uniquely frequent in pancreatic-cancer-involving deaths and that recent increases also occurred in lung and bronchus, colorectal, and liver and intrahepatic bile duct cancer deaths, without statistically significant differences in recent trends compared with pancreatic cancer. Substantial differences were observed by sex, race and ethnicity, and geography. These findings quantify death-certificate co-recording and do not establish the temporal sequence or causal contribution of renal failure. Linked cancer-registry, administrative, laboratory, treatment, and clinical data are needed to determine whether potentially modifiable kidney-related pathways contribute to these patterns.

## Figures and Tables

**Table 1:** Summary of deaths mentioning both pancreatic cancer and renal failure by demographic and geographic group, United States.

Group	Analytic Period	Deaths, *n*	First-Year AAMR (95% CI)	Final-Year AAMR (95% CI)	AAPC, % (95% CI)
Overall	1999–2024	31,497	0.37 (0.35–0.40)	0.82 (0.79–0.86)	3.47 (2.89–4.23)
Female	1999–2024	13,216	0.27 (0.24–0.30)	0.61 (0.57–0.65)	3.58 (3.13–4.45)
Male	1999–2024	18,281	0.55 (0.49–0.60)	1.03 (0.97–1.09)	2.97 (2.55–3.70)
Non-Hispanic Black or African American	1999–2024	5987	0.96 (0.80–1.12)	1.60 (1.46–1.76)	2.15 (1.57–2.78)
Non-Hispanic White	1999–2024	21,742	0.33 (0.30–0.36)	0.73 (0.70–0.77)	3.75 (3.26–4.42)
Hispanic or Latino	1999–2024	2403	0.43 (0.30–0.59)	0.58 (0.50–0.68)	1.34 (0.45–2.55)
Northeast	1999–2024	5758	0.40 (0.33–0.46)	0.75 (0.68–0.83)	2.60 (2.03–3.50)
Midwest	1999–2024	6871	0.42 (0.36–0.48)	0.82 (0.75–0.90)	3.49 (2.90–4.53)
South	1999–2024	11,457	0.35 (0.31–0.40)	0.83 (0.77–0.88)	3.53 (2.75–4.22)
West	1999–2024	7411	0.37 (0.31–0.43)	0.83 (0.76–0.90)	3.43 (2.77–4.67)
Metropolitan	1999–2020	19,097	0.39 (0.35–0.42)	0.55 (0.52–0.58)	2.00 (1.09–2.95)
Nonmetropolitan	1999–2020	3927	0.34 (0.28–0.41)	0.50 (0.43–0.57)	1.46 (0.40–2.87)

Note: AAMRs are expressed per 100,000 population and were standardized to the 2000 United States standard population. Urbanization analyses were limited to 1999–2020. The selected race and ethnicity categories do not sum to the overall total because other racial and ethnic categories are not displayed. AAMR, age-adjusted mortality rate; AAPC, average annual percent change; CI, confidence interval.

**Table 2:** Distribution and descriptive temporal changes in renal-failure subtypes among deaths mentioning both pancreatic cancer and renal failure, United States, 1999–2024.

Renal-Failure Subtype	ICD-10 Codes	Deaths, *n*	Percentage of Co-Recorded Deaths, % (95% CI)	1999 Deaths, *n*	1999 AAMR (95% CI)	2024 Deaths, *n*	2024 AAMR (95% CI)
Acute kidney failure	N17.0, N17.1, N17.2, N17.8, N17.9	13,612	43.22 (42.67–43.76)	142	0.09 (0.07–0.10)	1487	0.52 (0.49–0.54)
Chronic kidney disease	N18.0, N18.1, N18.2, N18.3, N18.4, N18.5, N18.8, N18.9	8818	28.00 (27.50–28.49)	141	0.07 (0.06–0.09)	670	0.23 (0.21–0.25)
Unspecified kidney failure	N19	9728	30.89 (30.38–31.40)	418	0.23 (0.21–0.25)	284	0.08 (0.07–0.09)

Note: Subtype categories represent any mention and are not mutually exclusive; percentages may therefore sum to more than 100%. AAMRs are expressed per 100,000 population and were standardized to the 2000 United States standard population. AAMR, age-adjusted mortality rate; CI, confidence interval.

## Data Availability

The mortality data analyzed in this study were obtained from the CDC WONDER Multiple Cause of Death, 1999–2020 and 2018–2024 datasets, available at https://wonder.cdc.gov/mcd.html (accessed on 26 June 2026).

## Supplementary Materials

The following supporting information can be downloaded at: https://www.mdpi.com/article/10.3390/epidemiologia7050124/s1, Table S1: Joinpoint regression estimates by demographic and geographic group; Table S2: Findings surrounding the transient elevation in deaths mentioning both pancreatic cancer and renal failure, United States, 2010–2013; Table S3: Comparison of renal-failure co-recording among deaths involving pancreatic cancer and selected major cancers, United States, 1999–2024; Figure S1: Annual age-adjusted mortality rates per 100,000 population for deaths mentioning pancreatic cancer and acute kidney failure, chronic kidney disease, or unspecified kidney failure, United States, 1999–2024.
