# Supplementary material for: Deaths Mentioning Both Pancreatic Cancer and Renal Failure in the United States, 1999–2024: Temporal Trends and Disparities
Source: Epidemiologia (Basel). 2026 Sep 4;7(5):124. doi: 10.3390/epidemiologia7050124 (PMC13600201; doi:10.3390/epidemiologia7050124)
Supplement: Supplementary file 1 [file epidemiologia-07-00124-s001.zip › epidemiologia-4416128-supplementary.pdf]

Supplementary Material

Supplementary Table S1. Joinpoint regression estimates by demographic and geographic group.

Group	Segment	Years	APC, %	95% CI	P value
Overall	1	1999–2011	2.90*	1.70 to 9.17	0.002
Overall	2	2011–2018	−1.46	−8.34 to 1.06	0.296
Overall	3	2018–2024	10.77*	7.18 to 18.22	0.006
Female	1	1999–2012	3.74*	2.58 to 6.33	<0.001
Female	2	2012–2015	−12.21*	−16.63 to −2.62	0.009
Female	3	2015–2024	9.21*	7.42 to 13.13	<0.001
Male	1	1999–2012	2.84*	1.79 to 5.38	<0.001
Male	2	2012–2016	−6.28*	−11.68 to −1.30	0.018
Male	3	2016–2024	8.15*	6.38 to 11.48	<0.001
Non-Hispanic Black	1	1999–2008	0.31	−7.35 to 5.04	0.909
Non-Hispanic Black	2	2008–2011	8.75	−5.46 to 13.25	0.081
Non-Hispanic Black	3	2011–2014	−11.63	−15.33 to 6.13	0.081
Non-Hispanic Black	4	2014–2020	2.08	−4.90 to 7.18	0.259
Non-Hispanic Black	5	2020–2024	13.32*	8.78 to 22.04	<0.001
Non-Hispanic White	1	1999–2011	3.91*	2.53 to 6.89	<0.001
Non-Hispanic White	2	2011–2016	−5.18*	−12.46 to −0.93	0.019
Non-Hispanic White	3	2016–2024	9.50*	7.50 to 12.41	<0.001
Hispanic or Latino	1	1999–2024	1.34*	0.45 to 2.55	0.007
Northeast	1	1999–2011	3.39*	2.09 to 6.49	0.002
Northeast	2	2011–2017	−4.83*	−14.36 to −0.79	0.014
Northeast	3	2017–2024	7.99*	4.89 to 16.92	<0.001
Midwest	1	1999–2012	2.57*	1.20 to 8.53	0.004
Midwest	2	2012–2016	−6.95	−14.69 to 0.68	0.085
Midwest	3	2016–2024	10.76*	7.88 to 17.60	0.012
South	1	1999–2020	1.72*	0.70 to 2.51	0.013
South	2	2020–2024	13.56*	7.00 to 26.75	<0.001
West	1	1999–2012	4.18*	2.73 to 8.59	0.002
West	2	2012–2016	−8.36*	−16.42 to −0.17	0.044
West	3	2016–2024	8.61*	5.74 to 17.27	0.002
Metropolitan	1	1999–2012	3.68	−0.23 to 5.93	0.052
Metropolitan	2	2012–2015	−8.78	−12.83 to 8.77	0.109
Metropolitan	3	2015–2020	4.53	−1.07 to 13.76	0.109
Nonmetropolitan	1	1999–2011	3.28*	1.76 to 15.54	0.010
Nonmetropolitan	2	2011–2020	−0.93	−9.13 to 1.24	0.393

Note: An asterisk indicates that the APC differed significantly from zero at $\alpha = 0.05$. APC, annual percent change; CI, confidence interval.

Supplementary Table S2. Findings surrounding the transient elevation in deaths mentioning both pancreatic cancer and renal failure, United States, 2010–2013.

Panel A. Overall, sex-specific, and Census-region-specific results

Group	2010 deaths, *n*	2010 AAMR	2011 deaths, *n*	2011 AAMR	2012 deaths, *n*	2012 AAMR	2013 deaths, *n*	2013 AAMR
Overall	1,096	0.51	1,327	0.60	1,388	0.62	1,062	0.45
Female	455	0.37	567	0.44	609	0.46	458	0.34
Male	641	0.65	760	0.80	779	0.80	604	0.59
Northeast	225	0.52	261	0.62	255	0.57	196	0.43
Midwest	212	0.43	290	0.60	297	0.58	224	0.42
South	397	0.48	464	0.56	470	0.55	401	0.49
West	262	0.56	312	0.67	366	0.73	241	0.46

Panel B. Percentage of pancreatic-cancer–involving deaths also mentioning renal failure

Year	Co-recorded deaths, *n*	All pancreatic-cancer–involving deaths, *n*	Co-recording percentage, % (95% CI)
2010	1,096	38,402	2.85 (2.69–3.03)
2011	1,327	38,862	3.41 (3.24–3.60)
2012	1,388	40,404	3.44 (3.26–3.62)
2013	1,062	40,659	2.61 (2.46–2.77)

Panel C. Renal-failure subtype results

Renal-failure subtype	2010 deaths, *n*	2010 AAMR	2011 deaths, *n*	2011 AAMR	2012 deaths, *n*	2012 AAMR	2013 deaths, *n*	2013 AAMR
Acute kidney failure	419	0.19	452	0.20	442	0.19	462	0.21
Chronic kidney disease	225	0.10	431	0.18	492	0.22	300	0.12
Unspecified kidney failure	461	0.22	468	0.21	478	0.22	319	0.13

Note: AAMRs are expressed per 100,000 population and were standardized to the 2000 United States standard population. Renal-failure subtype categories were based on any mention and were not mutually exclusive. AAMR, age-adjusted mortality rate; CI, confidence interval.

Supplementary Table S3. Comparison of renal-failure co-recording among deaths involving pancreatic cancer and selected major cancers, United States, 1999–2024.

**Cancer**	**Co-recorded / cancer deaths**	**Overall % (95% CI)**	**1999 %**	**2018 %**	**2024 %**	**APC 2018–2024, % (95% CI)**	**P vs pancreatic slope**
Pancreatic	31,497 / 1,057,018	2.98 (2.95–3.01)	2.26	2.77	4.33	9.15 (5.72–12.68)	Reference
Lung and bronchus	99,100 / 4,182,597	2.37 (2.35–2.38)	1.56	2.38	3.70	8.91 (5.68–12.23)	0.892
Colorectal	76,684 / 1,615,156	4.75 (4.72–4.78)	3.96	4.34	6.04	6.96 (4.39–9.59)	0.214
Liver/intrahepatic bile duct	35,676 / 618,504	5.77 (5.71–5.83)	4.42	5.42	7.88	7.79 (4.84–10.83)	0.438

Note: Co-recording percentage was calculated as deaths mentioning both the specified cancer and renal failure divided by all deaths mentioning that cancer. Fixed-period APCs for 2018–2024 were estimated using log-linear regression of annual co-recording percentages. P values compare each cancer-specific slope with the pancreatic-cancer slope using cancer-by-year interaction terms. APC, annual percent change; CI, confidence interval.

Supplementary Figure S1. Annual age-adjusted mortality rates per 100,000 population for deaths mentioning pancreatic cancer and acute kidney failure, chronic kidney disease, or unspecified kidney failure, United States, 1999–2024.

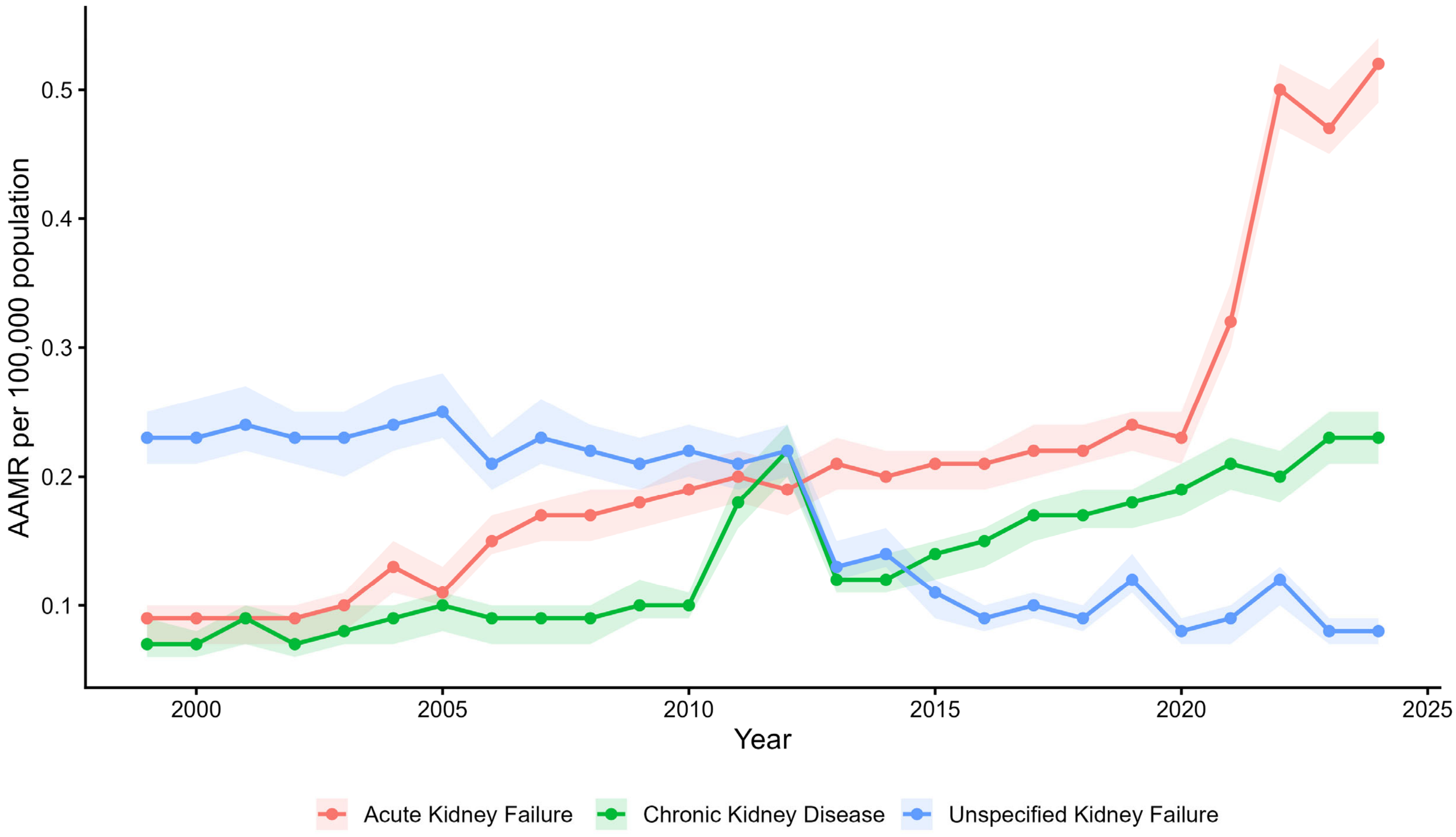

Note: Subtype categories were based on any mention and were not mutually exclusive. AAMR, age-adjusted mortality rate.
